# Hierarchical self-assembly in an RNA-based coordination polymer hydrogel†

**DOI:** 10.1039/d3dt00634d

**Published:** 2023-03-29

**Authors:** Osama El-Zubir, Pablo Rojas Martinez, Gema Dura, Cameron Doherty, Fabio Cucinotta, Lewis E. Mackenzie, Robert Pal, Benjamin R. Horrocks, Andrew Houlton

**Affiliations:** a Chemical Nanoscience Labs, Chemistry, School of Natural and Environmental Sciences, Newcastle University Newcastle upon Tyne NE1 7RU UK andrew.houlton@ncl.ac.uk; b Departamento de Química Inorgánica, Orgánica y Bioquímica, Facultad de Ciencias y Tecnologías Químicas UCLM Spain; c Department of Chemistry, Durham University South Road Durham DH1 3LE UK

## Abstract

An RNA-based coordination polymer is formed by the aqueous reaction of Cu^I^ ions with the thionucleoside enantiomer (−)6-thioguanosine, (6tGH). The resulting polymer, [Cu^I^(μ^3^-S-thioG)]_*n*_1, has a one-dimensional structure based on a [Cu_4_–S_4_] core and undergoes extensive hierarchical self-assembly transforming from oligomeric chains → rod → cable → bundle through which a fibrous gel forms, that undergoes syneresis to form a self-supporting mass. The assembly involves the formation of helical cables/bundles and, in combination with the intrinsic photoemission of the polymer, results in the material exhibiting circularly polarised luminescence (CPL).

## Abstract 2

An RNA-based coordination polymer is formed by the aqueous reaction of Cu^I^ ions with the thionucleoside enantiomer (−)6-thioguanosine, (6tGH).

## Graphical abstract

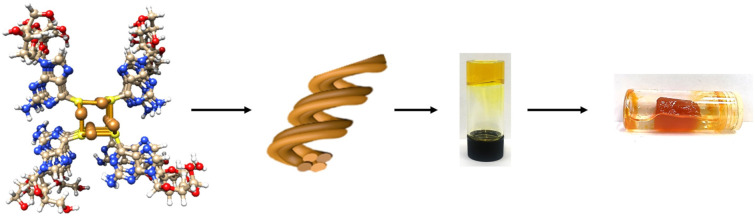

## Introduction

Coinage-metal thiolate (CMT) polymers,^1^ [M^I^(μ-SR)]_*n*_, are a class of materials that exhibit a range of useful opto-electronic properties such as tunable luminescence,^2–4^ non-linear optics^5^ and electrical conductivity^6–9^ on account of their d^10^ metal configuration, extended delocalization^6^ and even band-like electronic structure.^7^ Atomically-precise structural details are available for these compounds containing all the monovalent group 11 metal ions with the majority forming one-dimensional coordination chains, which may exhibit helicity.^1^ The processability of these, often insoluble, materials can be problematic requiring use of high temperatures and/or multiple stages. In response, recent efforts have been made to address this including the use of mechanical pressure to form transparent glasses^10^ and polymer formation at modified surfaces.^11^

We have been exploring thio-modified nucleosides, such as the thiopurine 6-thioguanosine ((−)6-tG), to develop new types of functional coordination motifs from nucleic acid-based components. Notably, we have shown these can form the [M^I^(μ-SR)]_*n*_ coordination chains observed in this class of compounds due to the metal-ion-bridging capacity of the sulfur group.^6,12^ Compared to the natural nucleosides, this minimal O → S modification profoundly alters the metal-ion specificity, available binding mode and, consequently, the physicochemical properties compared to canonical nucleoside complexes.^6,12,13^ In addition, we have also found that these compounds readily form as hydrogels and so offer new possibilities in terms of processivity, ease of manipulation and potential application for this class of materials. Here, we report on the synthesis and characterization of cuprous 6-thioguanosine, [(Cu(i)-μ-S-(−)6tG)_*n*_], 1, a coordination-chain polymer that displays a highly ordered hierarchical self-assembly to form a hydrogel that exhibits syneresis – the expulsion of previously trapped solvent from the gel matrix upon its contraction.^14^ The material exhibits large chiro-optics on account of the helical nature of the resulting assemblies and luminescence in both normal and circularly polarized forms.

## Results and discussion

### Cu(i)-6-thioguanosine, 1, preparation and characterisation

**Fig. 1:**
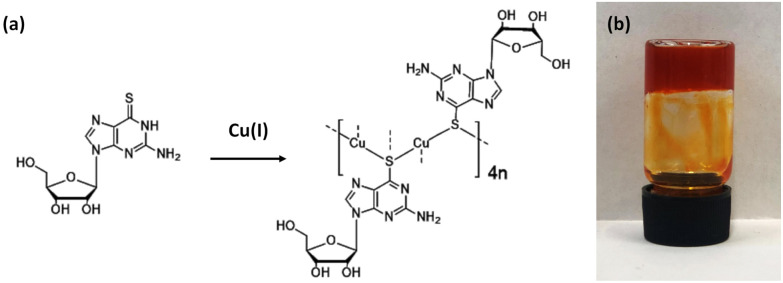
(a) Reaction scheme for the formation of Cu(i)-thioguanosine 1 and (b) optical image of the hydrogel at concentration of 30 mmol l^−1^ in a glass vial.

The reaction of equimolar equivalents of Cu(i) ions, as [Cu(MeCN)_4_]PF_6_, with an aqueous dispersion of the sulfur-modified nucleoside enantiomer (−)6-thioguanosine, (6tG-H), forms a viscous yellow solution within 1 hour. Over the course of ∼5–7 days the reaction mixture transforms into a gel, as assessed by the inversion tube test, and continues to darken in colour until, after four weeks, a dark orange hydrogel is formed (Fig. 1).

The product of the reaction is shown to be the corresponding one-dimensional copper-thiolate coordination polymer, [(Cu(i)-μ^3^-S-(−)6tG)_*n*_], 1. Changes upon Cu–S binding are seen in the FTIR indicating thione (C=S) → thiolate (C–S^−^) conversion as a loss of the ∼1200 cm^−1^ absorption and appearance of bands at ∼680 cm^−1^ corresponding to the latter (see ESI Fig. 1†). The polymeric nature is revealed by MALDI-MS with peaks corresponding to the following oligomers: [Cu_3_L_3_]^+^ (found (*m*/*z*) 1085.2; calc. 1084.97) [Cu_4_L_3_]^+^ (found (*m*/*z*) 1148.1; calc. 1147.9), [Cu_5_L_4_]^+^ (found (*m*/*z*) 1511.0 calc. 1510.89). [L corresponds to a deprotonated anionic thiolate form of 6-thioguanosine, [C_10_H_12_N_5_O_4_S]^−^] (see ESI Fig. 2†). In the UV-visible spectrum, the longer wavelength absorption band of the parent nucleoside at ∼339 nm, with band edge extending to about 380 nm, is red-shifted upon binding Cu(i) to ∼356 nm and broadened with the band edge reaching 500 nm (ESI Fig. 3†). XPS shows the binding energy of Cu 2p_3/2_ peak is 932.7 eV indicating the +1 formal oxidation state. Furthermore, the presence of Cu^2+^ valence state can be excluded as the Cu 2p_3/2_ shake-up satellite peak of Cu^2+^ at binding energy 943 eV is absent from the spectrum (ESI Fig. 4†).^15,16^

**Fig. 2:**
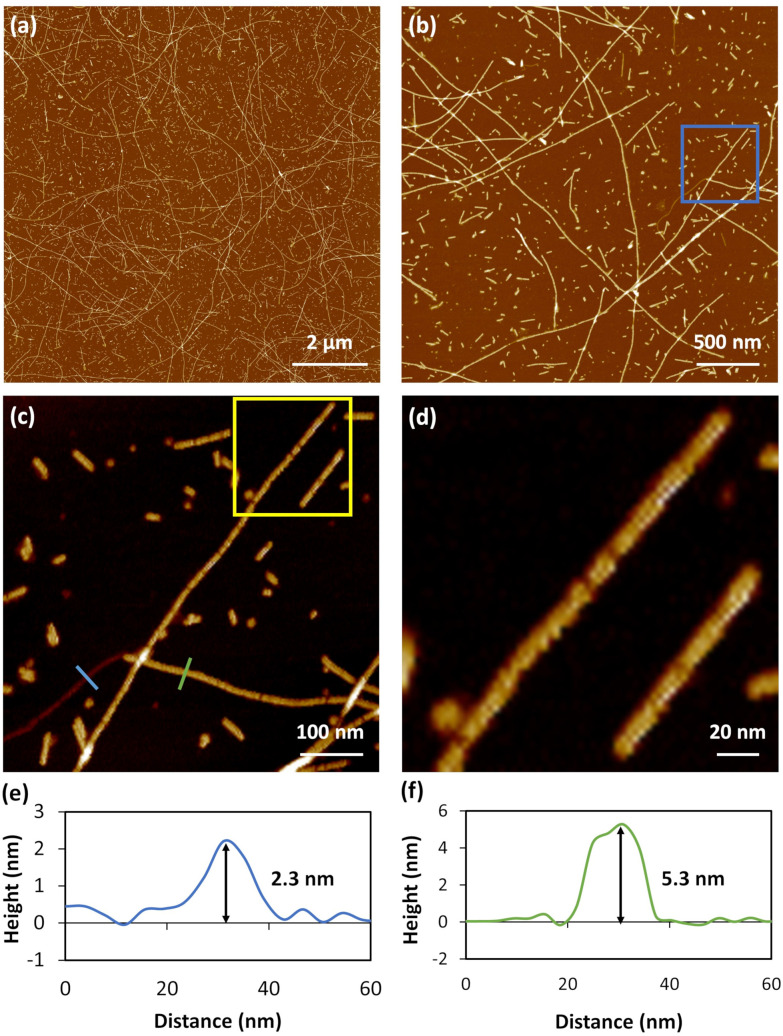
AFM topographical images of Cu-thioguanosine xerogel drop-cast onto a mica surface. (a and b) Large scan range image. (c) Zoom-in on the blue box in image (b). (d) Zoom-in the yellow box in image (c) showing the helical structure of individual rods. The associated cross-sections along the blue (e) and green (f) lines in the AFM image (c).

AFM analysis of freshly prepared samples reveals a one-dimensional structure for 1. Large-area scans show that the coordination polymer strands extend several microns in length and are homogenous in height (Fig. 2). The main features in samples measure 5.35 ± 0.32 nm in height (Fig. 2c and f) and show relatively little curvature over 100 s of nm. It can be noted, however, that there are also some strands that are smaller in height, at 2.26 ± 0.28 nm (Fig. 2c and e) which are the smallest features observed.

**Fig. 3:**
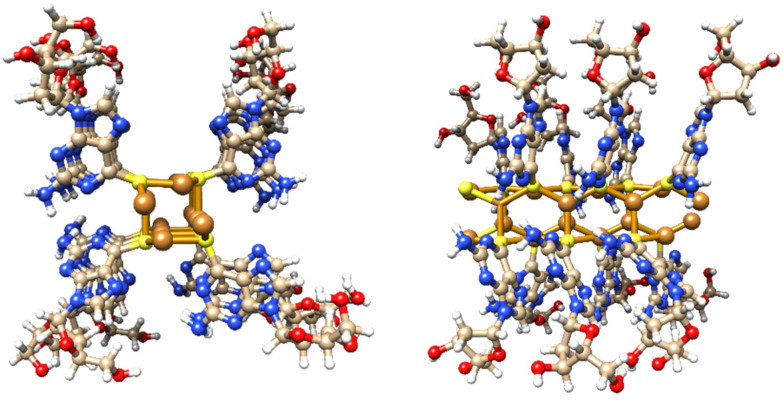
Views of a model of the Cu_4_S_4_-core rod structure of 1 which has an approx. diameter of 2.4 nm and involves μ^3^-bridging 6-thioG.

Single crystal X-ray diffraction data for cuprous-thiolate polymers show two distinct structure types,^1^ as either single {Cu(i)-μ-SR-}_*n*_ “chains” or tetrameric {Cu(i)-μ^3^-SR-}_*n*_ “rods”; the latter essentially containing 4 “chains” crosslinked by μ^3^-bridging of the thiolate group in contrast to simple two-metal bridged “chains”. Models of 1 indicate a single {Cu(i)-μ-SR-}_*n*_ “chain” structure to have a diameter <2 nm. In contrast, a [{Cu(i)-μ^3^-SR}_4_]_*n*_ “rod” has a diameter ∼2.4 nm, *closely matching the height of the smallest features observed in the AFM images*. From this we propose that 1 has a basic Cu_4_S_4_-core “rod”-type structure as shown in Fig. 3.

**Scheme 1:**
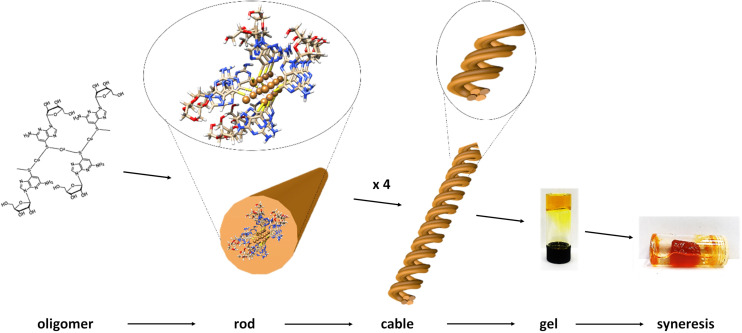
Hierarchical self-assembly from coordination oligomer to gel of 1. The proposed stages involve initial short, single-chain, oligomer formation with further coordinate-bond-driven assembly into rods. Individual rods assemble through non-covalent, *e.g.* aromatic stacking, interactions into helical cables which entangle to form the fibrous-gel network. Further assembly drives contraction of the gel matrix and exclusion of solvent, syneresis.

However, given the small fraction of these ∼2.3 nm rods observed in a typical AFM image, these must rapidly assemble into the observed dominant structural features, with height ∼5.3 nm. We denote these larger features as “cables” that comprise several “rods”. A noticeable feature of the “cables” is the evident twist along the main axis, consistent with helicity. All individual “cables” show a right-handed sense, with a pitch of ∼20 nm, as can be seen in Fig. 2d. Based on the size of the features in the AFM and simple models we suggest that an individual cable is assembled from four rods twisted into a helical structure through a simple braiding arrangement. The persistence of these features in samples suggests that this is a highly favourable configuration and is a basic unit of the material in the subsequent assembly towards the gel state. These “cables” further aggregate into larger fibre bundles and entangle to form the gel network. Scheme 1 illustrates the proposed stages of this hierarchical self-assembly process from coordination oligomers to a fibrous gel network that displays syneresis (see Fig. 5 and Scheme 1).

**Fig. 4:**
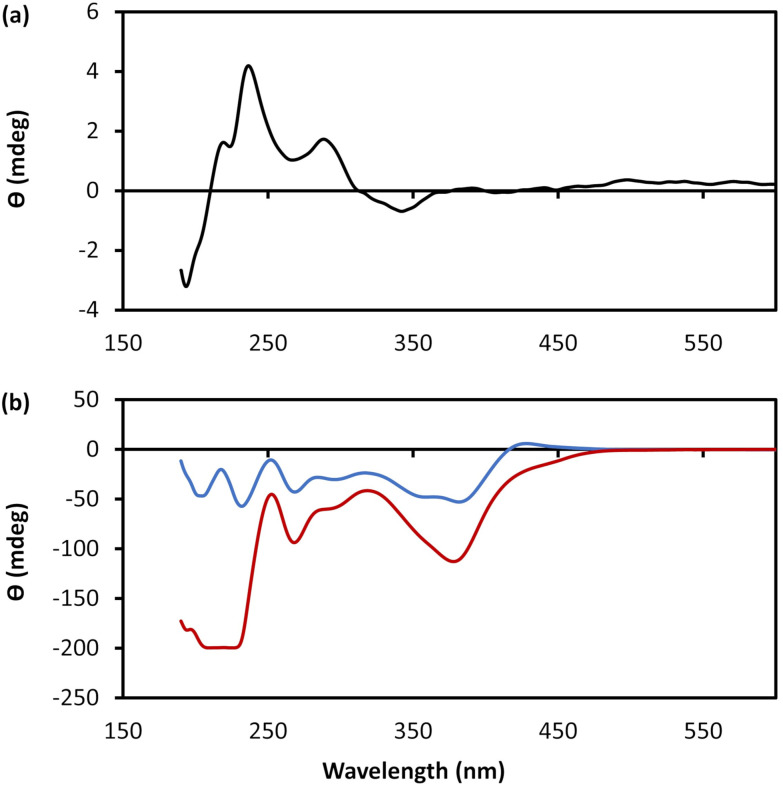
CD spectra. (a) CD spectrum of an aqueous solution of 6-TGH. (b) CD spectra of an aqueous solution of 1 at a concentration of 10 mmol l^−1^ as-prepared (blue) and after 28 days (red).

Additional confirmation of the helicity of 1 was provided by CD and, compared to the ligand itself, this shows significantly more intense bands across the UV-visible range with enhancement of (at least) one order of magnitude (Fig. 4 and ESI Fig. 5†). Such an amplification in the chiro-optics is attributed not only to the coordination polymer structure ordering the chiral nucleoside chromophore but also to the assembly being directed to a single helical form.^17^ Notably, the appearance in the CD spectrum of a band at *ca*. 380 nm, which can be attributed to metal–ligand charge transfer of the polymer backbone, indicates some degree of helical twist of the central {Cu_4_–S_4_} coordination unit. [Note: such helicity is not indicated on the short rod model in Fig. 3.]

### Hydrogel formation, ageing and evolution of optical properties

**Fig. 5:**
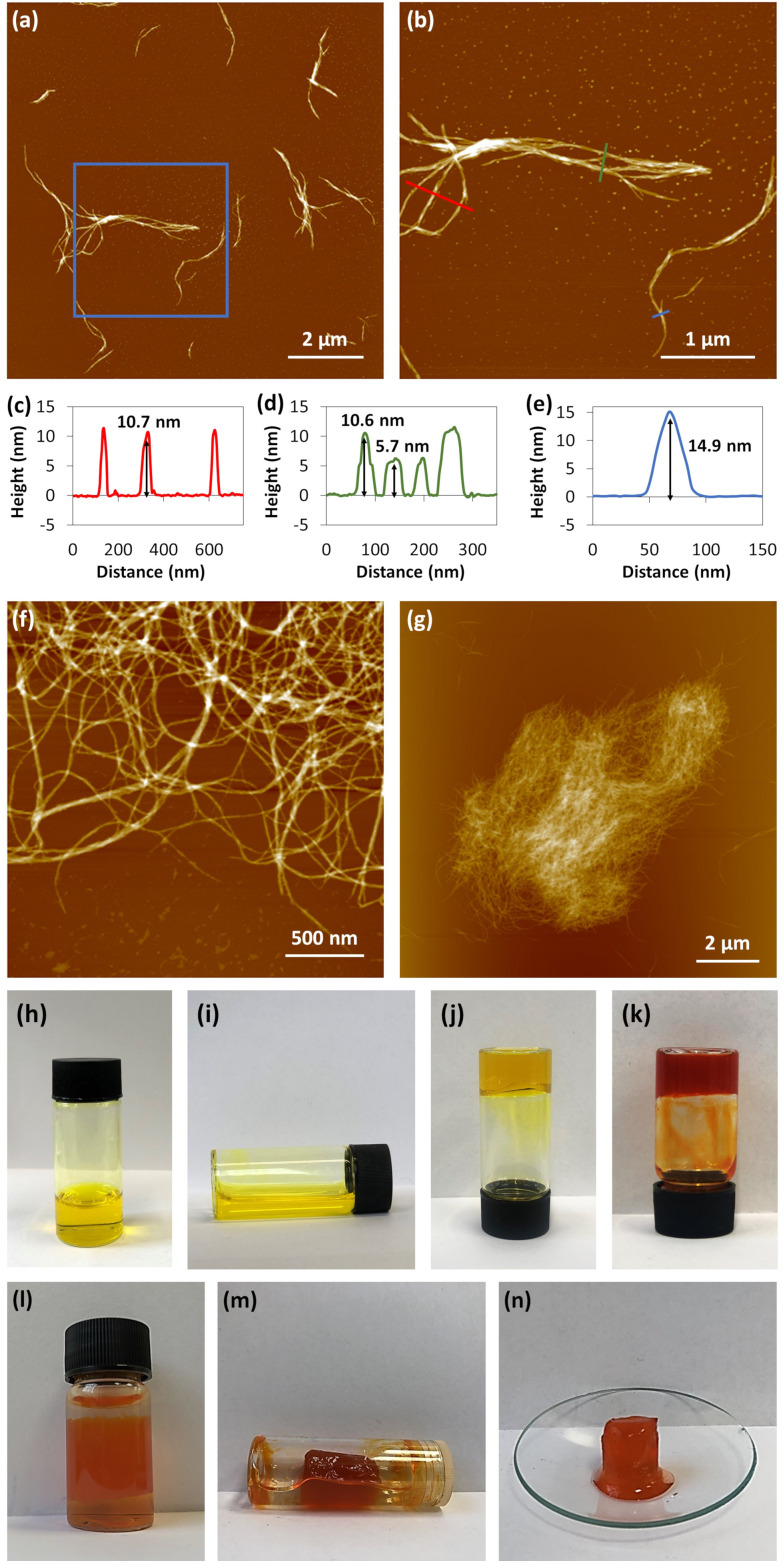
AFM topographical images of xerogel of 1 drop-cast onto a mica surface. (a) Large scan range image. (b) Zoom-in on the blue box in image (a). The associated cross-sections along the red (c), green (d) and blue (e) lines in the AFM image (b). (f) An image showing the bundled and tangled polymer chains of 1. (g) A cluster of aged hydrogel that has been diluted in water and dried on mica. Optical images of (h and i) a fresh, (j) 9 days and (k) 6 months old sample hydrogel at concentration of 30 mmol l^−1^ in a glass vial. (l) An optical image of the hydrogel at concentration of 10 mmol l^−1^ in a glass vial starts to expulse water. (m) The hydrogel at concentration of 10 mmol l^−1^ in a glass vial after the syneresis process. (n) Self-supporting gel body removed from water after syneresis.

The gelation of 1 is a slow process compared to the other coinage metal analogues^6,12^ and, uniquely for this series of compounds, displays syneresis^14^ with the resulting gel-body sufficiently mechanically robust to be self-supporting (Fig. 5n). Upon initial reaction, rapid complexation is indicated by the formation of a yellow, viscous, solution (Fig. 5h–i). The reaction mixture continues to transform and, over a period of 5–7 days, forms a hydrogel (at concentration of 30 mmol l^−1^) (Fig. 5j). The hydrogel continues to darken in colour to form, after ∼4 weeks, a final orange/brown hydrogel (Fig. 5k) which is remarkably stable (>3 months).

**Fig. 6:**
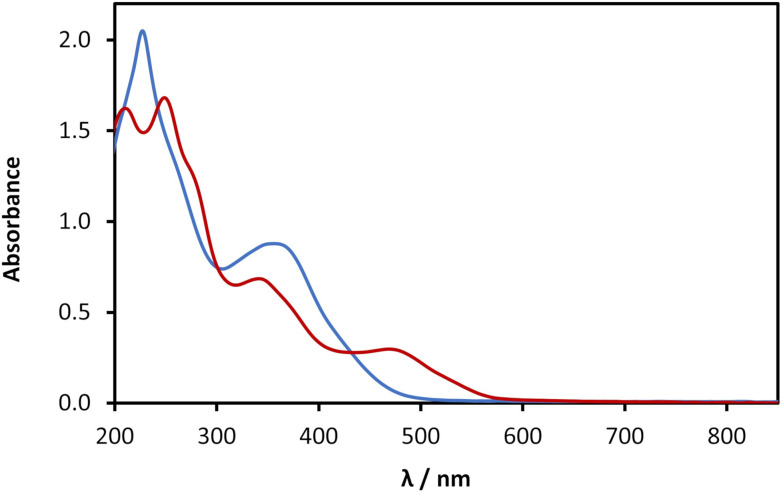
UV-Vis absorption spectra of a fresh solution (blue) and aged solution (red) of 1. Both solutions were prepared at concentration of 30 mmol l^−1^ and diluted to 1 mmol l^−1^.

This transformation upon ageing can be followed by UV-vis absorption spectroscopy (Fig. 6) with the spectra showing a decrease in intensity of the ∼350 nm region with time (Fig. 6). Furthermore, a new peak at ∼470 nm is observed, and the band edge extends to ∼580 nm after about 4 weeks of preparing the polymer. We ascribe this to the further self-assembly of 1 and the emergence of charge transfer transitions within the polymer chains.

AFM images of gels after ageing for several weeks (Fig. 5a and b) continue to highlight a basic one-dimensional nature though assembly continues with the formation of fibres. This is shown in Fig. 5a–e which shows cross-section heights of different parts of a fibrous network as 10.6 nm and 14.9 nm. These measure as approximate multiples of the basic “cable” structure further illustrating that this assembly is a favourable, stable, arrangement. These fibres become entangled (Fig. 5f), as expected in the process of forming the gel network.

Rheological measurements using oscillatory sweep tests (ESI Fig. 6†) confirmed that the critical concentration to form stable gels is 30 mmol l^−1^; concentrations below this did not form stable gels, possibly on account of low degrees of entanglement of a less flexible “cable” structure. Rheological assessment using oscillatory sweep tests confirmed the slow process of gelation. For fresh samples, the storage modulus value was around 10 Pa. This value increased with time from 55 Pa for 5 days to 100 Pa for 4 weeks (ESI Fig. 7†). In all cases the storage modulus was higher than loss modulus. *G*′ represents the elastic property and is a measure of the magnitude of the recoverable energy that is stored in the material. The *G*′′, on the contrary, is a measure of the energy that is lost as viscous dissipation. With time of ageing, the differences between both these parameters increased (ESI Fig. 6†), indicating the hydrogel developed a more solid-like structure. Frequency sweep experiments (ESI Fig. 8†) showed *G*′ was larger than *G*′′ across the range of frequencies studies (0.1–100 rad s^−1^), indicating the elastic nature of the gel. However, for fresh samples G′ showed more frequency dependence, and this dependence decreased with the age of the gel, suggesting the predominance of the elastic response with time, due to an increase in inter-polymer interactions. This is consistent with the formation of a more solid-like material and denser, well-formed, network structure.

The linear viscoelastic region of the gel was evaluated using oscillatory studies (ESI Fig. 9†) where *G*′ is independent of strain applied for this concentration indicating the structure of the gel is not disrupted and remains intact. The linear viscoelastic region decreased dramatically with ageing, from 40% for fresh preparations, 10% after 5 days and 6.5% for 4 weeks after reaction. Furthermore, the critical strain was found when *G*′ = *G*′′. For gels of 1 this value decreased with the time, indicating that the gel becomes less flexible with the ageing, from 364% for fresh samples to below 100% after 4 weeks. The viscosity was studied under shear rate experiments (ESI Fig. 10†), for fresh samples and after 1 week. Both fresh and 1-week old samples showed a shear-thinning behaviour, reducing the viscosity by 2- (1 week) or 3-orders of magnitude (fresh) allowing easier flow with shear rate. The backward experiment suggested that the disruption of the network by shear can be recovered instantaneously. Interestingly, it was not possible to perform the same experiments with gels >4 weeks old since these broke into fragments after the application of shear force.

As mentioned above, an interesting difference between hydrogels of 1 compared to those of the Au(i)^6^ and Ag(i)^12^ analogues is that 1 shows syneresis.^14^ This is seen as contraction of the gel network over time with associated expulsion of solvent and the resulting gel body becoming self-supporting (Fig. 5l–n). In supramolecular hydrogels this process generally occurs through increased fibre-fibre interactions which are influenced by hydrophobicity.^14^

### Photoluminescence and circularly polarised luminescence

**Fig. 7:**
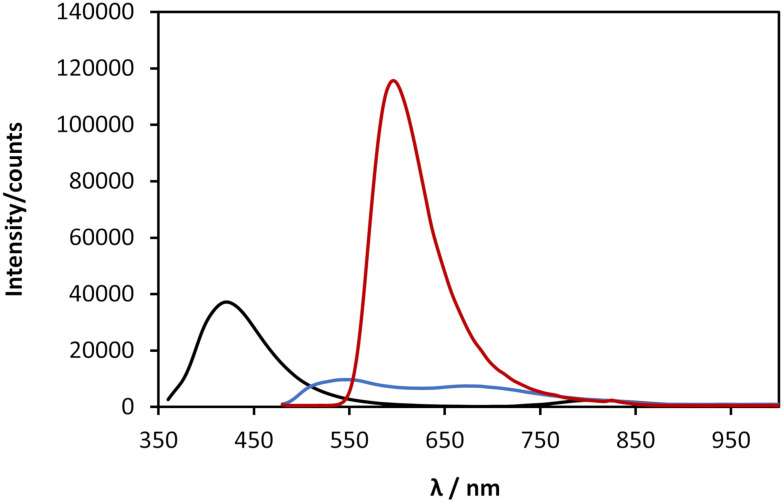
Fluorescence emission spectrum of a solution of 6-TGH nucleoside at a concentration of 30 mmol l^−1^ in 0.1 mol l^−1^ of NaOH (black), excitation at 350 nm, and emission spectra of a solution of 1 at concentration of 30 mmol l^−1^ as it is fresh (blue) and after gelation (red), excitation at 470 nm.

Coinage-metal thiolate polymers are recognized as luminescent materials,^1–3,9^ and this is true for 1 which exhibits room temperature luminescence. This luminescent emission is significantly shifted into the red region of the visible spectrum compared to that of the free ligand (*λ*_Em_ 420 nm) (Fig. 7, black line). In the case of freshly prepared samples the polymer exhibits a broader emission spectrum, with a maximum ∼513 nm and a minor band after 650 nm (Fig. 7, blue line). After ageing, 1 undergoes gelation and a colour change, as shown in Fig. 5, and with this the emission spectrum shows a red-shifted band at 600 nm, accompanied by a large increase of the luminescence intensity (Fig. 7, red line). Such a change upon ageing is consistent with the appearance of a new absorption peak at 470 nm (Fig. 6) and the consequent shift in the absorption band edge (Fig. 6 and ESI Fig. S3†).

As indicated earlier (see Scheme 1), we attribute this to the self-assembly of 1 from rods to thicker bundles. Such further order of assembly rigidifies the system and significantly reduces the energy losses by non-radiative decay, causing a much-enhanced luminescence compared to the parent ligand and the fresh product. The continued assembly is supported by the AFM imaging (Fig. 5). Previously reported copper(i) thiolate coordination polymers showed enhanced and red-shifted emission bands upon cooling, as an effect of the increased rigidity,^10^ and we ascribe the optical behaviour of 1 to such an effect, which in our case is brought about by the formation of close-packed rods/bundles from single chains. Also in this case,^10^ the red-shifted emission appearing over the 550–750 nm range is attributed to mixed MLCT and metal cluster-centred CT transitions.

**Fig. 8:**
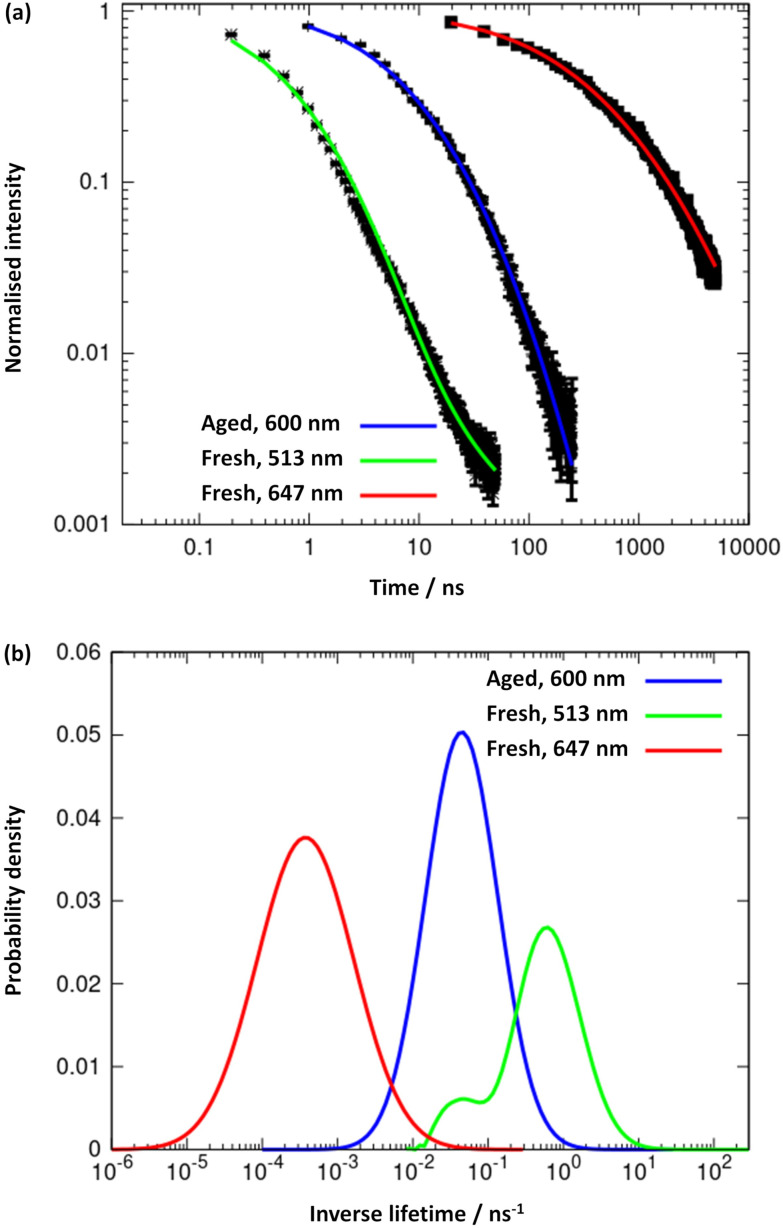
(a) Logarithmic plots and trendlines of the excited-state decay profiles, recorded at 513 nm (trendline in green) and 647 nm (red line) for the fresh sample, and at 600 nm (blue line) for the aged one; excitation was performed at 371 nm. (b) Distribution of excited-state lifetimes extracted from the fits to the decay curves.

The results obtained from the time-resolved luminescence measurements complement the above attribution and add further insights. The excited-state decay profiles recorded for the freshly-prepared sample at 513 nm and at 647 nm, under excitation at 371 nm, are markedly different (Fig. 8a, green and red lines, respectively). None of the decays are single-exponentials but can be fitted to a distribution of lifetimes (Fig. 8b), for which we quote the modal values. A short lifetime of 1.6 ns is observed when the decay is monitored at 513 nm and a much longer lifetime of 2.8 ms is observed for the decay at 647 nm. The latter is well-fitted by a lognormal distribution of lifetimes. We suggest that the lower energy state is a charge-transfer state and the higher energy state is ligand-centred. In aged samples, water is excluded by syneresis and a single photoluminescence band is observed at about 600 nm (Fig. 7). The lifetime of this is much shorter than that of the charge-transfer state in the fresh sample and was measured as 21.3 ns, suggesting a ligand-centred transition (see also ESI Fig. 11–13†). We interpret the data in terms of changes to the solvation of the structure: a charge-transfer state is likely to be destabilised by the exclusion of polar solvent molecules and therefore not populated in the aged sample to the same extent as in the fresh sample. This is further supported by the reduction of the FWHM with ageing, as the 600 nm emission band is narrower than the 647 nm band found in the fresh sample. Thus, the increased rigidity and the lack of solvent stabilisation upon ageing are at the origin of the lifetime reduction.

**Fig. 9:**
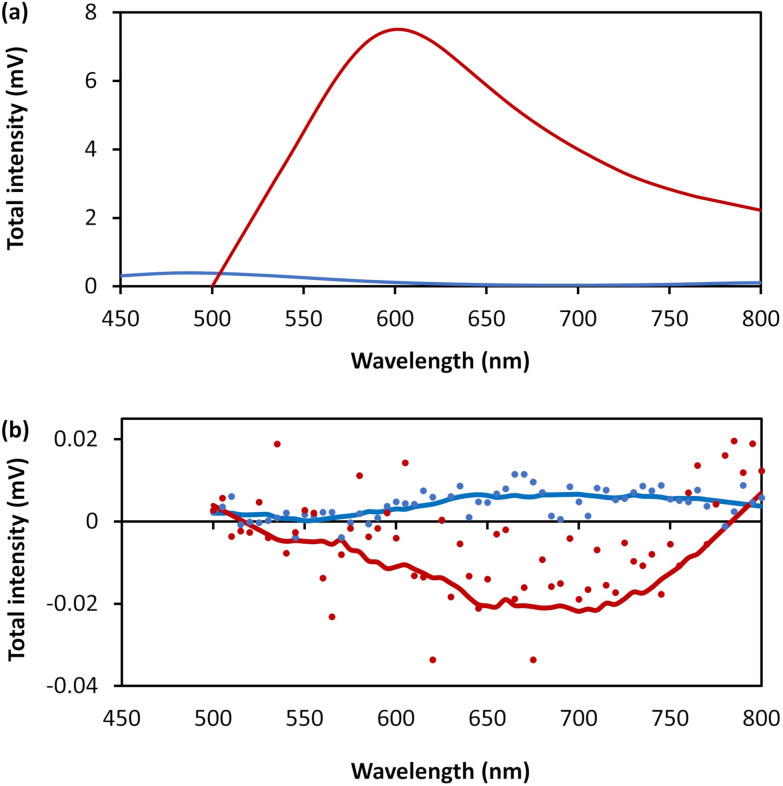
(a) Total photoluminescence spectra of 6-thioguanosine at a concentration of 30 mmol l^−1^ in 0.1 mol l^−1^ of NaOH (blue) and 1 at concentration of 20 mmol l^−1^ (red). (b) CPL raw spectra of the 6-thioguanosine (blue dots), the 1 (red dots), the smoothed spectrum of the 6-thioguanosine (blue line) and the smoothed spectrum of 1 (red line).

The combination of intrinsic emissive properties and helicity of the assembled polymer rod/bundle gives rise to circularly polarised luminescence (CPL) (Fig. 9). The luminescence dissymmetry factor (*g*_lum_) for 1 (20 mmol l^−1^) is −1.17 × 10^−2^ at 720 nm. As noted previously, solutions of 6-thioguanosine show no significant CPL.^12^ The CPL emission of 1 can be explained by a combination of the delocalization of the excited state in the rigid, helical, self-assembled structure with possible contribution from chiral scattering effects due to the handedness of the gel fibres, as discussed for Ag^I^-6-thioguanosine gels.^12^

## Conclusions

We have recently shown that CPL is displayed by Ag^I^-6-thioguanosine, the first example of a CMT-polymer to do so.^12^ Our reporting here of similar behaviour for the Cu^I^ derivative, 1, highlights that this may be a quite general feature of this broad class of materials if prepared, or fabricated, in appropriate forms. Moreover, the ability of all the group 11 metals, Cu, Ag,^12^ and Au,^6^ to form [M^I^-μ-(6-thioguanosine)]_*n*_ polymers illustrates similarities in terms of coordination chain formation, helical architecture in their assemblies and the formation of entangled networks suitable for solvent trapping/hydrogel formation. However, the compounds show subtle, and not so subtle, differences in the detailed self-assembly at both a coordination and supramolecular bond level. Copper-containing 1 is unique in having an [M_4_S_4_] core and also shows a more extensive hierarchical assembly process than the heavier congeners, with the resulting gel state, uniquely, undergoing syneresis. Together, these compounds provide a series of metallo-hydrogel materials displaying useful optical and mechanical properties and, valuably, extends the range of physico-chemical properties available for CMT-coordination polymers.^1^

## Conflicts of interest

There are no conflicts to declare.

## Supplementary Material

DT-052-D3DT00634D-s001
