# Supplementary material for: Hierarchical self-assembly in an RNA-based coordination polymer hydrogel
Source: Dalton Trans. 2023 Mar 29;52(17):5545–51. doi: 10.1039/d3dt00634d (PMC10152458; doi:10.1039/d3dt00634d)
Supplement: DT-052-D3DT00634D-s001 [file DT-052-D3DT00634D-s001.pdf]

Electronic Supplementary Material (ESI) for Dalton Transactions.
This journal is © The Royal Society of Chemistry 2023

Supplementary Information

Hierarchical self-assembly in an RNA-based coordination polymer hydrogel

Osama El-Zubir,[1] Pablo Rojas Martinez,[1] Gema Dura,[2] Cameron Doherty,[1] Fabio Cucinotta,[1] Lewis E. Mackenzie,[3] Robert Pal,[3] Benjamin R. Horrocks,[1] Andrew Houlton[1]

[1] *Chemical Nanoscience Labs, Chemistry, School of Natural and Environmental Sciences, Newcastle University, Newcastle upon Tyne, NE1 7RU, U.K.*
[2] *Departamento de Química Inorgánica, Orgánica y Bioquímica, Facultad de Ciencias y Tecnologías Químicas, UCLM, Spain.*
[3] *Department of Chemistry, Durham University, South Road, Durham, DH1 3LE, UK.*
[*] *Corresponding author. Email: andrew.houlton@ncl.ac.*

Table of contents

1. METHODS 2
1.1 Materials 2
1.2. Preparation of **1** 2
1.3. FTIR spectroscopy 2
1.4. Matrix-assisted laser desorption/ionization Spectrometry (MALDI-Tof) 2
1.5. Ultraviolet-Visible Spectroscopy (UV-Vis) 2
1.6. X-ray photoelectron spectroscopy 2
1.7. Atomic Force Microscopy (AFM) 2
1.8. Circular dichroism 2
1.9. Rheology 2
1.10. Fluorescence spectroscopy 2
1.11. Excited-state lifetime measurements 2
1.12 Circularly polarized luminescence (CPL) 3
1.13 Modelling........ 3
2. Supplementary Figures 4
3. References 14

1. Methods

1.1. Materials. All reagents were purchased from Sigma-Aldrich. Deionised water (18 MΩ cm resistivity) was obtained from a Direct-Q® 3 UV Water Purification System (Merck).

1.2. Preparation of 1. Samples of Cu^{I}-thioguanosine, $[(Cu(I)\text{-}\mu^3\text{-}S\text{-}(\text{-})6tG)_n]$ **1**, as a hydrogel were prepared as follows. An aqueous suspension of 6-thioguanosine (6TG-H) was sonicated for 30 min to achieve a fine dispersion. A molar equivalent weight of copper(I) tetrakis(acetonitrile) hexafluorophosphate was added to the suspension of 6TG-H. After the addition of the Cu(I) ion, the mixture was stirred magnetic stirrer for an hour. A yellow solution is formed which becomes more viscous over time, transforming after ~28 days into an orange gel.

1.3. FTIR spectroscopy. An IRAffinity-1S Fourier transform infrared spectrophotometer (Shimadzu) equipped with an ATR accessory has been used to record FTIR spectra in the range of 400 to 4000 cm^{-1} wavenumbers at 4 cm^{-1} spectral resolution. Each spectrum was generated by co-adding and averaging 64 scans. The bare ATR accessory served as the background. Prior to analysis, a hydrogel 1 sample was deposited onto a clean p-Si(100) chip (1 cm2) and air-dried for one day. (Supplementary Fig. 1)

1.4. Matrix-assisted laser desorption/ionization Spectrometry (MALDI-Tof). A Bruker Autoflex II ToF/Tof Mass Spectrometer was used to record the MALDI spectra. To prepare the sample, hydrogel **1** (60 mmol l^{-1}) was diluted 100-fold in ultrapure water, and then 1 µl of the diluted solution was mixed with 9 µL of an acetonitrile solution of α-cyano-4-hydroxycinnamic acid as the matrix.

1.5. Ultraviolet-Visible Spectroscopy (UV-Vis). Absorption spectra between 190 and 850nm were recorded in a NanoDrop™ OneC UV-Vis spectrophotometer. UV-Vis spectra of aqueous solutions of 6-thioguanosine at concentrations of 1 mmol l^{-1} and of a fresh and aged solution of **1** at concentrations of 1 mmol l^{-1} were recorded in a quartz cuvette with pathlength 2 mm. The spectrometer was blanked using nanopure water.

1.6. X-ray photoelectron spectroscopy. The Thermo Scientific K-Alpha X-ray photoelectron spectrometer (Thermo Electron Corp., East Grinstead, UK) with an Al Ka X-ray source (1486.6 eV) was utilized to obtain XPS spectra. A take-off angle of 90° was employed during data acquisition, and a charge neutralization gun used to compensate for surface charging. High-resolution spectra of the individual core orbitals were acquired at a pass energy of 50 eV and 0.1 eV/step. The XPS spectra were analyzed and curve-fitted using CasaXPs software (Casa, http://www.casaxps. com, UK). All spectra were corrected to hydrocarbon C1s peak at binding energy of 285 eV as reference.

1.7. Atomic Force Microscopy (AFM). Multimode 8 atomic force microscope with a NanoscopeV controller (Bruker), and an "E" scanner was used to obtain AFM data. The microscope was controlled using Nanoscope software version 9.1, and operated in ScanAsyst in Air mode as a peak force tapping mode at ultra-low forces minimise damage to the samples. To reduce vibrational noise, an isolation table/acoustic enclosure (from Veeco Inc., Metrology Group) was employed. Imaging was done using silicon tips on silicon nitride cantilevers (ScanAsyst, Bruker), with a nominal tip radius of approximately 2 nm, resonant frequency of 150 kHz, and spring constant k ~0.7 Nm^{-1}. The AFM data were analyzed with NanoScope Analysis 1.5 software (Bruker). To prepare the sample, 2 µl of freshly prepared dilute aqueous solution of 1 was added onto a mica surface and dried in air.

1.8. Circular dichroism. Circular dichroism spectra were recorded on a Jasco J-810 Jasco J-810 Spectropolarimeter. CD spectra of a solution of an aqueous solution of 6-thioguanosine and **1** (both at concentration of 10 mM) were recorded in a quartz cell with pathlength 0.1 mm.

1.9. Rheology. Rheological measurements were performed with a HR-2 Discovery Hybrid Rheometer (TA Instruments) with a standard steel parallel-plate geometry of 20 mm diameter with a gap of 1 mm. The strain and the frequency were set to 1% and 1 Hz, respectively.

1.10. Fluorescence spectroscopy. Emission spectroscopy was recorded on a SPEX Fluoromax spectrofluorimeter. Emission spectra were recorded on a solution of 6-thioguanosine at concentration of 30 mmol l^{-1} in 0.1 mol l^{-1} of NaOH, excitation at 350 nm. Emission spectra of **1** were measured on a solution at concentration of 30 mmol l^{-1} (as-prepared and after gelation), excitation at 470 nm. The spectra were recorded in a quartz cuvette with a path length of 0.2 cm.

1.11. Excited-state lifetime measurements. Luminescence spectra and excited state lifetimes were measured using an Edinburgh FLS980 photoluminescence spectrometer, equipped with a 450W Xenon arc lamp, Czerny Turner excitation and emission monochromators (1.8 nm/mm dispersion; 1800 grooves/mm), time-correlated single photon counting (TCSPC) module and a Hamamatsu R928 P photomultiplier tube (in a fan assisted TE cooled housing, operating temperature −20°C). Emission spectra were recorded on a solution of 6-thioguanosine (1 and 30 mmol l^{-1}) in 0.1 mol l^{-1} of NaOH. Emission spectra of **1** were measured on a solution at concentration 1 mmol l^{-1} and a gel at concentration 30 mmol l^{-1}. The spectra were recorded in a quartz cuvette with a path length of 0.2 cm. For lifetime measurements, samples were excited with an EPL-375 (370.8 nm; 61.1 ps pulse width) and an EPL-475 (471.8 nm; 61.1 ps pulse width) picosecond pulsed diode lasers and data analysis was performed on the F980 software with numerical data reconvolution based on Marquardt-Levenberg algorithm.

1.12 Circularly polarized luminescence (CPL). CPL spectra were recording using a custom-build CPL spectrometer. Full details of this CPL spectrometer have been reported by Carr et al.[1] Non-standard protocols were followed to measure CPL from the gel samples. Total emission and CPL emission were sampled at 400 - 800 nm in 5 nm increments, with 10 accumulated spectra per measurement. Roughly 500 micro-liters of the gel sample was contained in an open-topped quartz cuvette (101-10-40, Hellma). The gel adhered to a corner of the cuvette. Excitation was provided by a 410 nm laser source from directly above the sample. The laser and sample were positioned to maximise emission intensity from the sample. Data was processed using custom-written Matlab scripts (Matlab 2019b, Mathworks). Instrumental baselines for total emission intensity and CPL emission were subtracted to zero measurements as appropriate and were then smoothed using an intensity-preserving Savitzky-Golay filter. g_{lum} values for each repeated measurement were calculated from smoothed intensity and smoothed CPL emission.

1.13 Modelling. Spartan 16 ver. 2.0.9 (Wavefunction Inc.) was used for model building and figures produced using UCSF Chimera 1.11.2.

2. Supplementary Figures

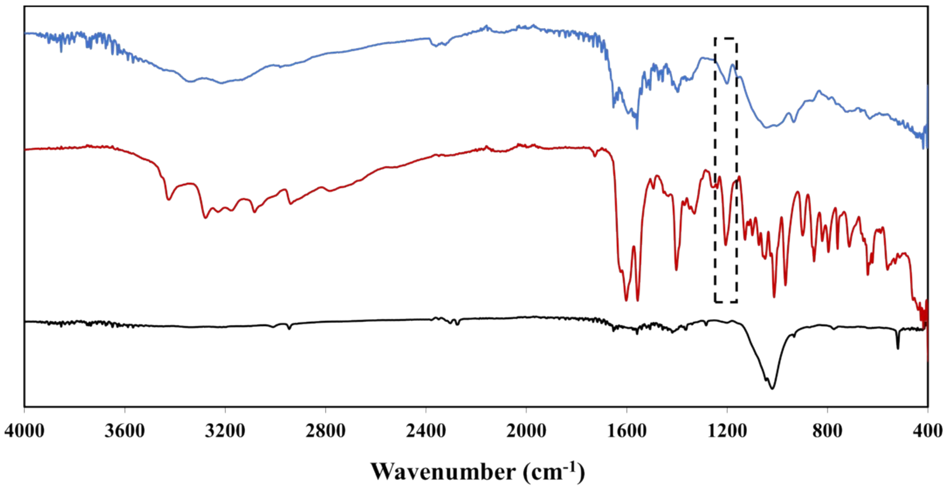

Supplementary Figure 1. FTIR spectrum of **1** xerogel (blue), 6-thioguanosine (red) and $Cu(MeCN)_4BF_4$ (black). The band indicated at ~1200 cm^{-1} is attributed to (C=S) in 6-thioguanosine.

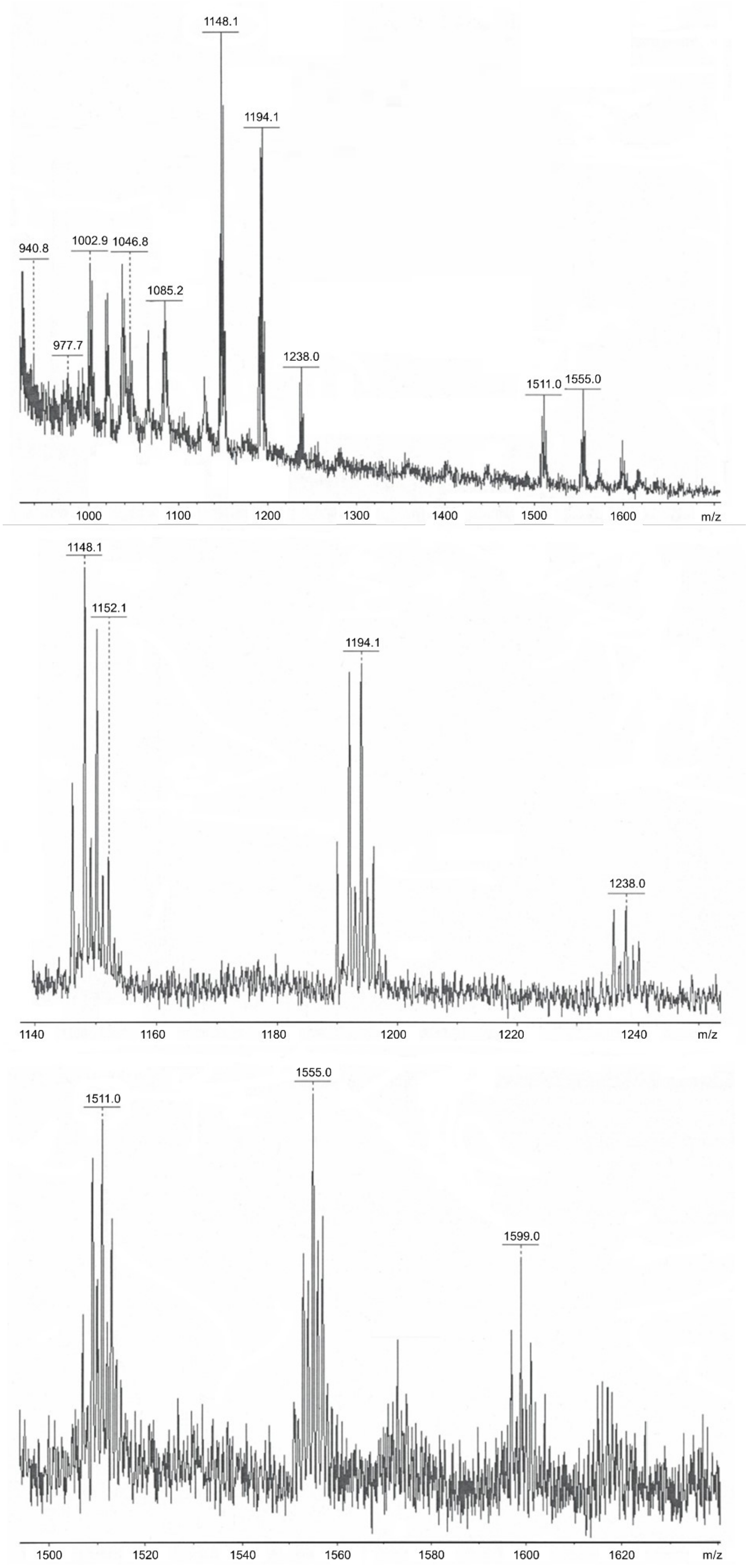

Supplementary Figure 2. MALDI-MS data measured for **1**.

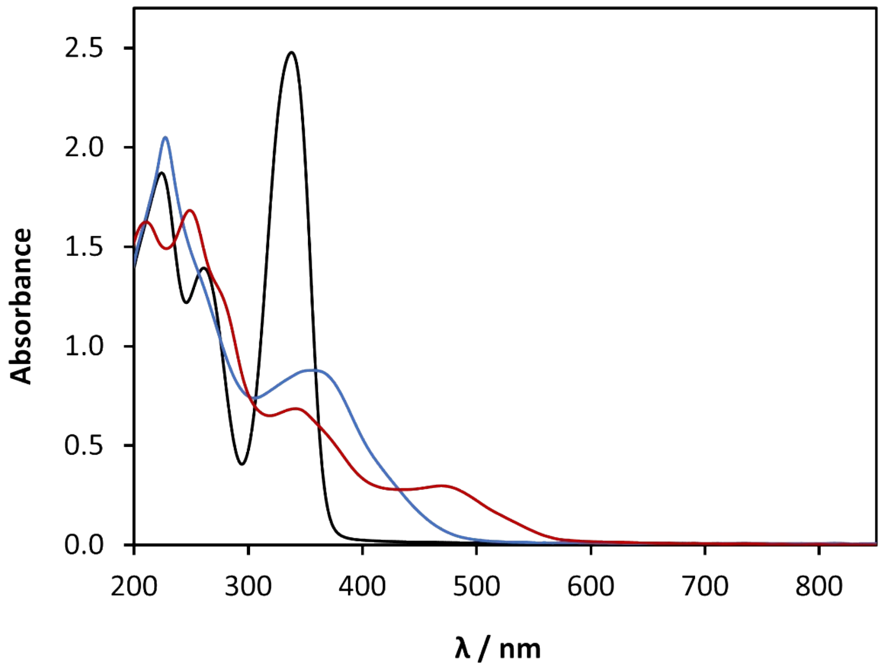

Supplementary Figure 3. UV-Vis absorption spectrum of an aqueous solution of 6-TGH at a concentration of 1 mmol l^{-1} (black), and absorption spectra of a fresh solution (blue) and aged solution (red) of **1**. Both solutions of **1** were prepared at concentration of 30 mmol l^{-1} and diluted to 1 mmol l^{-1}.

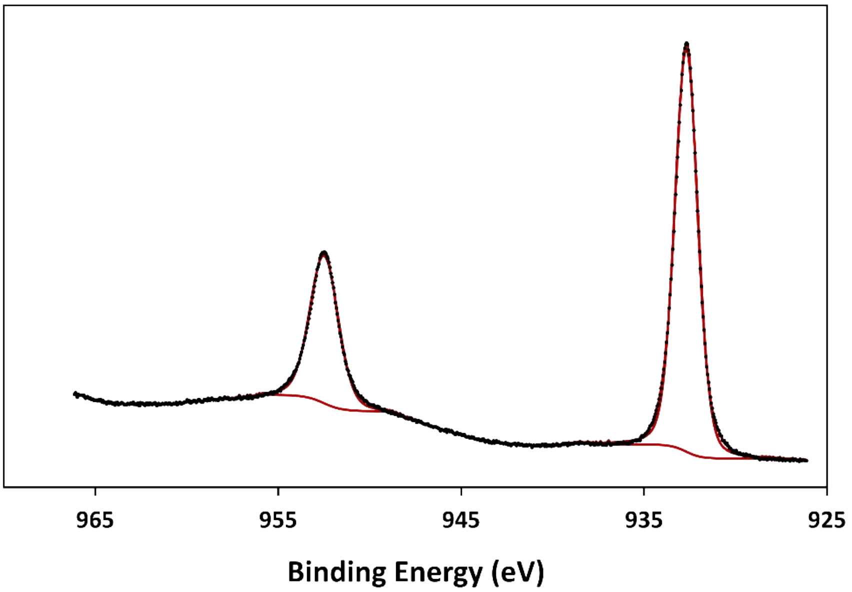

Supplementary Figure 4. High-resolution XPS core level spectrum of Cu $2p_{1/2}$ and $2p_{3/2}$ region that was recorded on xerogel **1**.

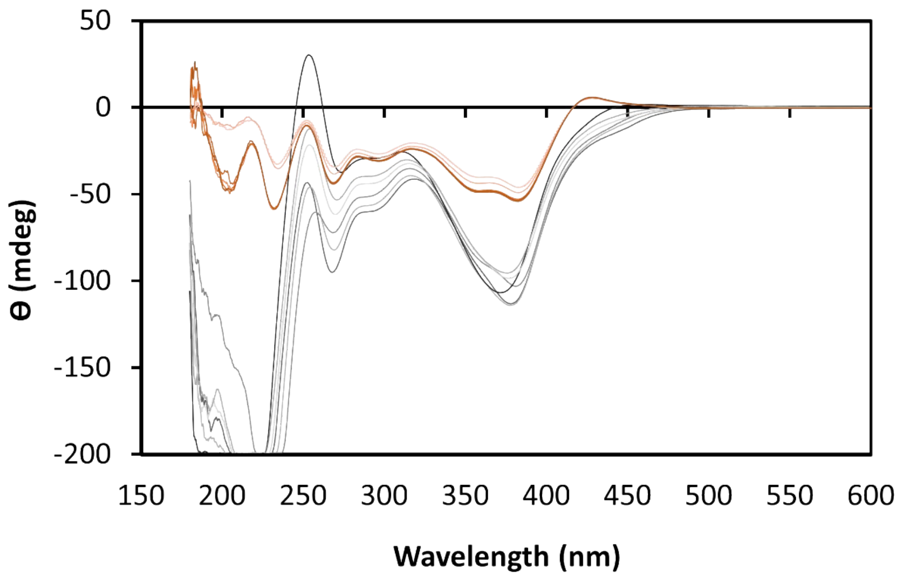

Supplementary Figure 5. CD spectrum of solutions of **1** at a concentration of 10 mmol l^{-1} when they are fresh (orange) and after 28 days/4 weeks (black). These spectra are from 6 experimental repeats.

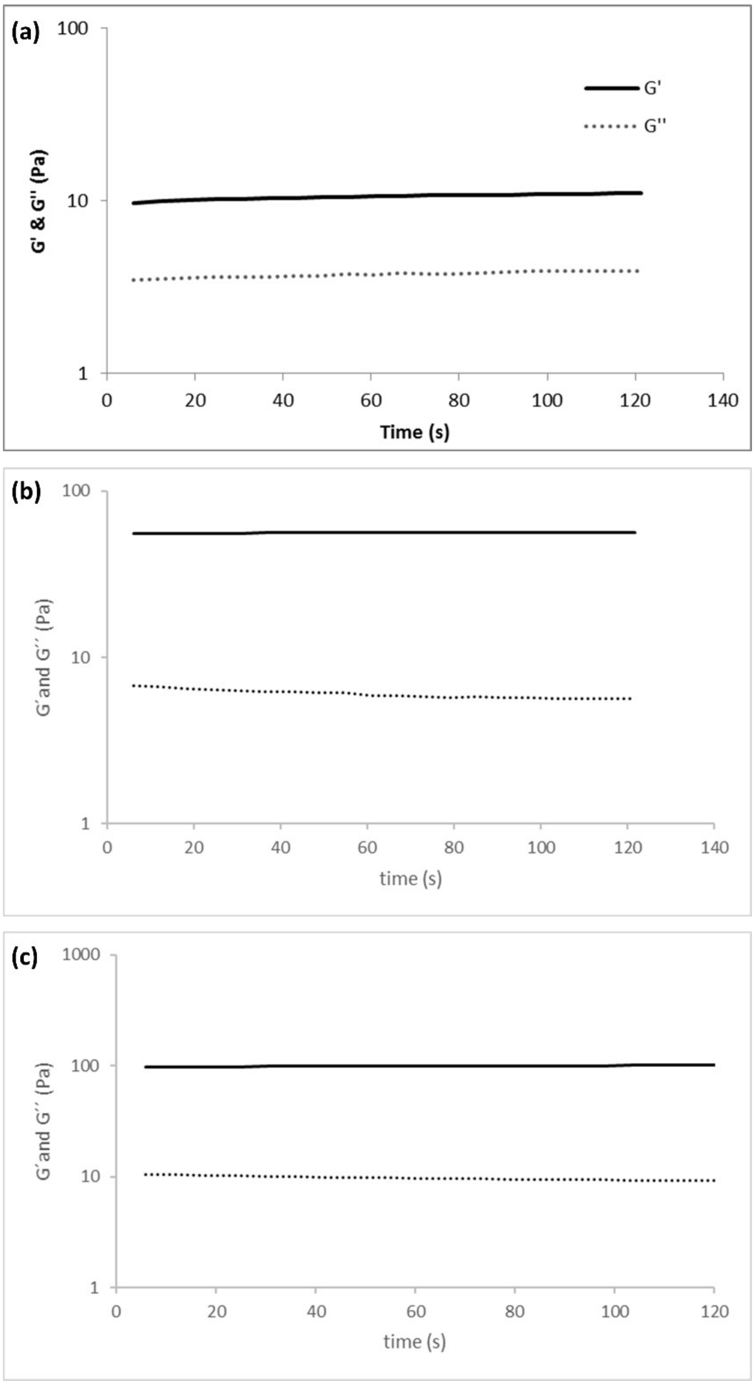

Supplementary Figure 6. Time sweep experiments of hydrogels of **1** at 30 mM. (a) Fresh sample, (b) 5 days of gelation and (c) 4 weeks of gelation.

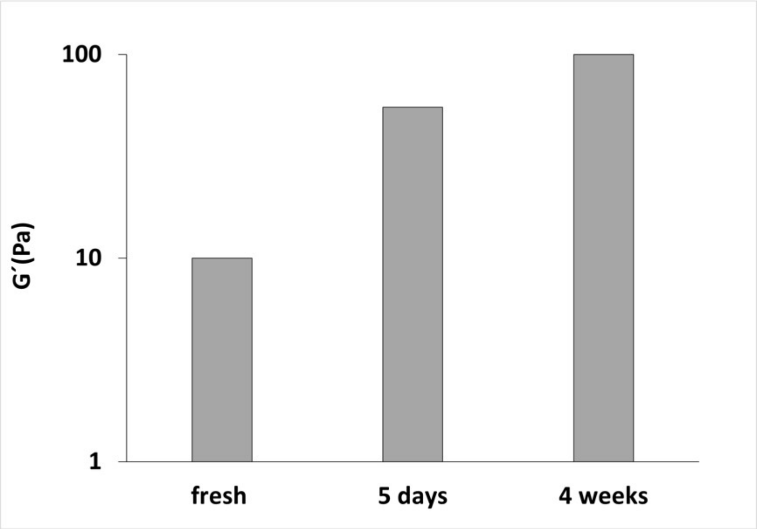

Supplementary Figure 7. G´values of samples of hydrogels of **1** at 30 mM at different times of gelation.

(a)

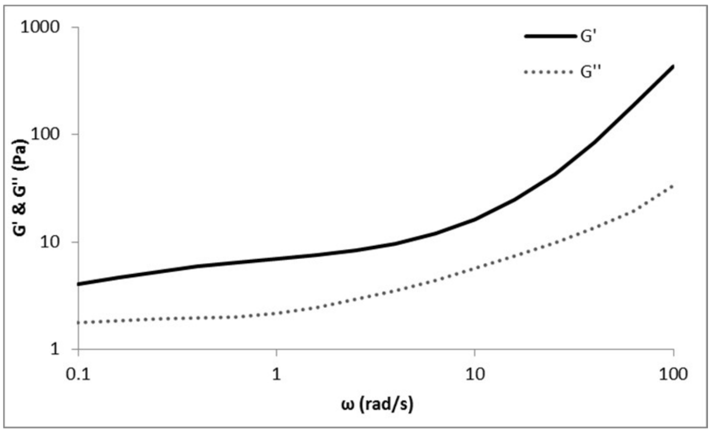

(b)

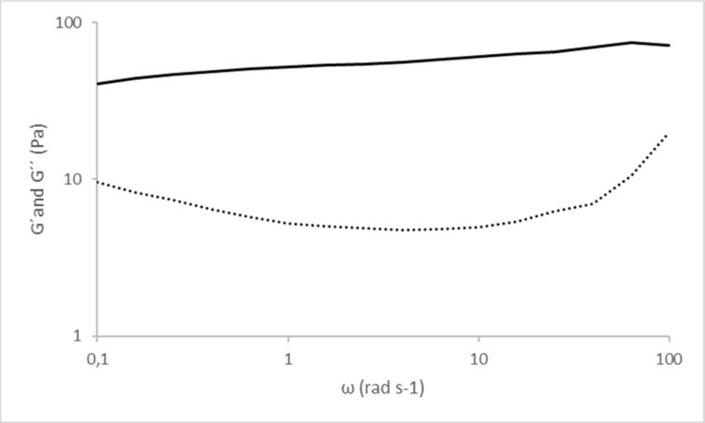

(c)

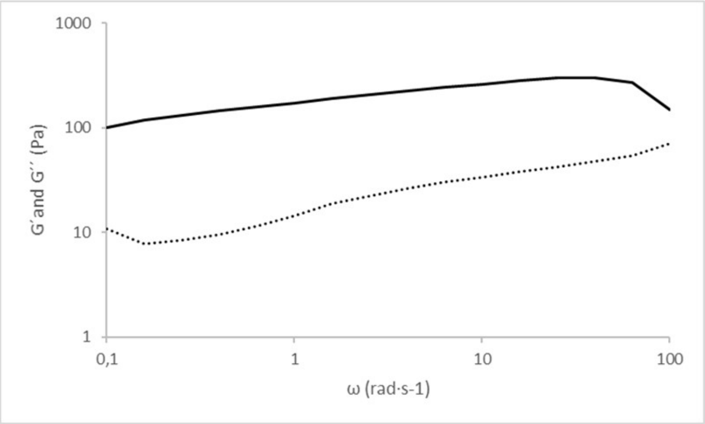

Supplementary Figure 8. Frequency sweep experiments of hydrogels of **1** at 30 mM. (a) Fresh sample. (b) 5 days of gelation. (c) 4 weeks of gelation.

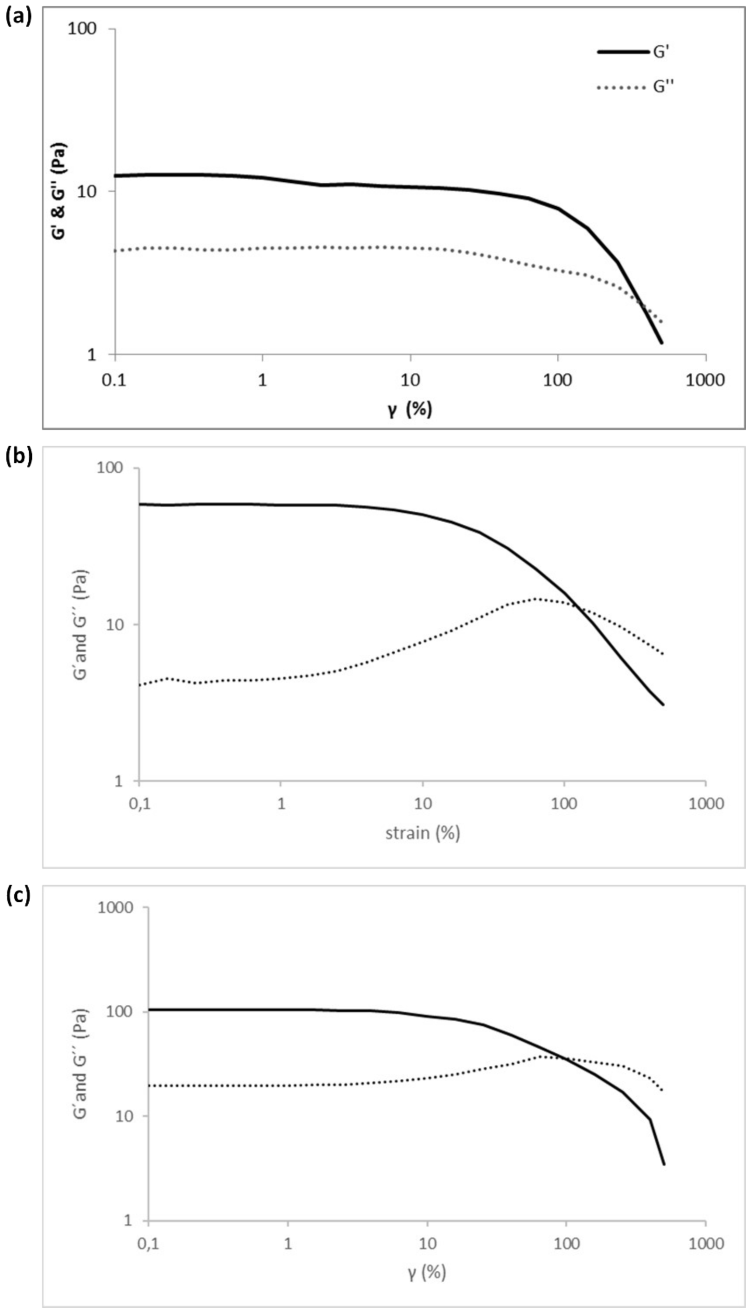

Supplementary Figure 9. Strain sweep experiments of hydrogels of **1** at 30 mM. (a) Fresh sample. (b) 5 days of gelation. (c) 4 weeks of gelation.

(a)

(b)

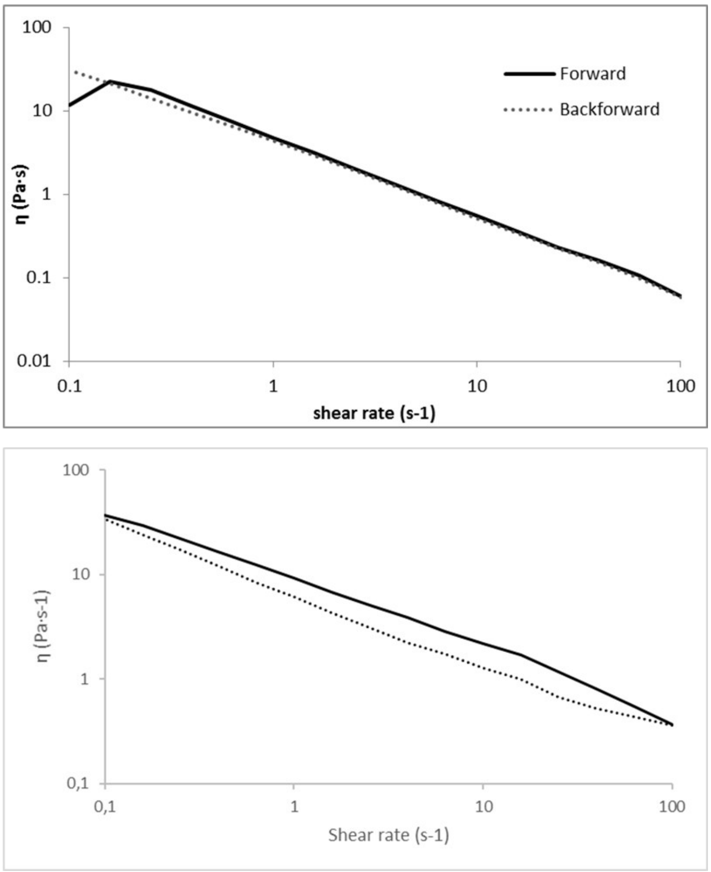

Supplementary Figure 10. Shear rate experiments of hydrogels of **1** at 30 mM. (a) Fresh sample. (b) 5 days of gelation.

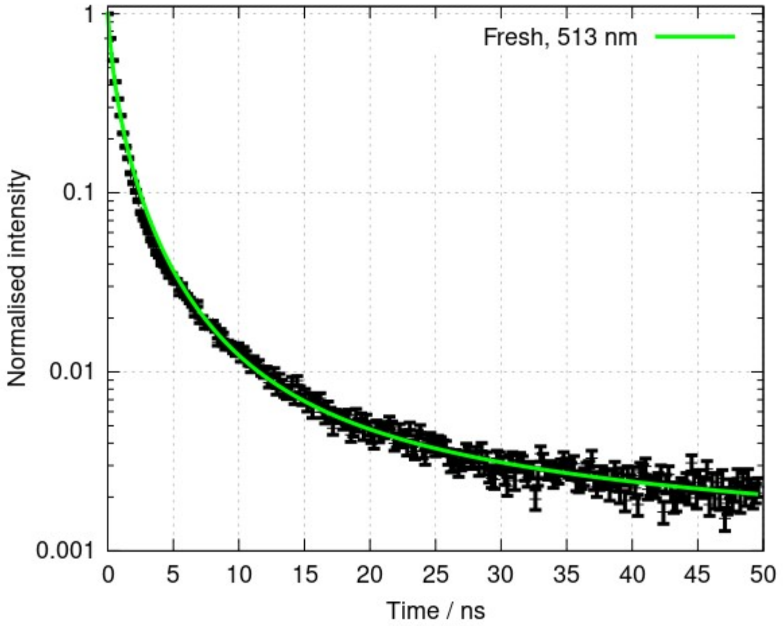

Supplementary Figure 11. Semi-logarithmic plot of the excited-state decay profile of fresh hydrogel **1**, recorded at 513 nm using a 371 nm laser.

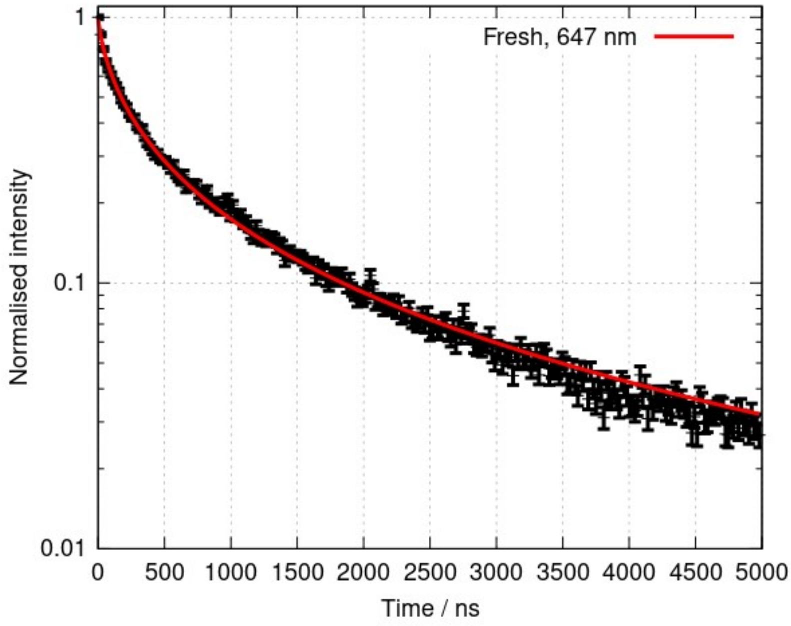

Supplementary Figure 12. Semi-logarithmic plot of the excited-state decay profile of fresh hydrogel **1**, recorded at 647 nm using a 371 nm laser.

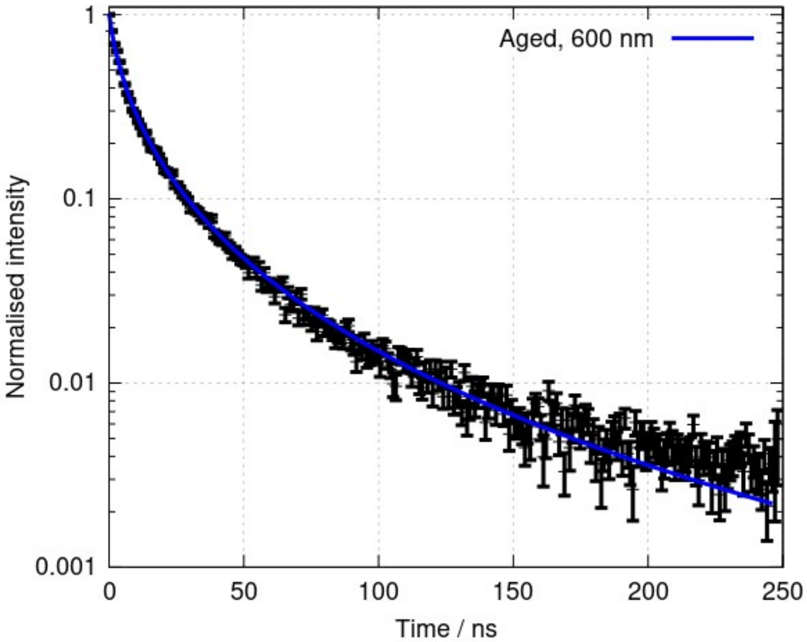

Supplementary Figure 13. Semi-logarithmic plot of the excited-state decay profile of the aged hydrogel **1**, recorded at 600 nm using a 371 nm laser.

References

1. R. Carr, R. Puckrin, B. K. McMahon, R. Pal, D. Parker and L. O. Palsson, *Methods Appl. Fluoresc.*, 2014, **2**.
